# Tincture of Perchloride of Iron in Post Partum Hemorrhage

**Published:** 1882-10-20

**Authors:** J. H. Scarff

**Affiliations:** Baltimore


### TINCTURE OF PERCHLORIDE OF IRON IN POST-PARTUM HEMORRHAGE.

BY J. H. SCARFF, M.D., BALTIMORE.

(Cases related at the Medical and Surgical Society)

It is not my purpose to enter into a discussion of the general treatment of post partum hemorrhage, nor do I wish to be understood as placing this agent among the first to be used to combat these hemorrhages, but as a dernier ressort when all others have failed and your patient seems to have but one more step to realize the mysteries of the hereafter. Many of you, no doubt, have read the many able articles of European writers upon this subject of late, and probably have had more experience in its application, but having had two cases in the past four months of alarming hemorrhage following labor, and as I confidently believe my patients owe their prolonged sojourn in this land of sin and sorrow to the injection of perchloride of iron into the cavities of their uteri, I do not hesitate to relate the two following cases in which I have used this remedy:

I wish it to be distinctly understood, I claim no originality to either the agent used or the modus operandi in using it. It originated with that eminent obstetrician, Barnes of London, and several cases so treated by him were published in the British Medical Journal of 1876 or '77. Following the report of his cases, there came other testimonials to the virtue of this agent, from Playfair, Chambers, Steele and others. As I have before stated, this potent agent should not be used indiscriminately in all cases, but only after such remedies as ergot, external pressure, cold, kneading of the uterus and galvanism have failed.

CASE I. Mrs. M——, wife of a druggist (which was a most fortunate circumstance for her), was taken in her third labor on the afternoon of February 11th. I had previously ascertained from her husband that her first two labors were followed by most alarming hemorrhages, but through the skill of the lamented Dr. Knight she was saved. Nothing worthy of mention occurred during the first stage of labor excepting a prolonged interval of quiescence between the pains. This stage lasted about $4\frac{1}{2}$ hours. After delivery I immediately cut the cord, as it had ceased to pulsate. Turning my attention to the woman, I found the uterus as large as before the birth of the child, with a soft and doughy feeling. Introducing my hand into the vagina I found the placenta was detached and blocking up the os uteri; it was at once removed, and with it there came a most appalling hemorrhage, and in a very short space of time the woman was completely blanched, pulse almost imperceptible, sighing respiration and other symptoms indicating an almost complete collapse. I hurried her husband down into his store for a bottle of tincture of perchloride of iron. I diluted it one-half with water, and with the aid of a Davidson's syringe which I had procured, this was injected into the cavity of the uterus. (I would state I first, as well as I could, emptied the uterus of all clots.) The effect was magical; almost directly after the injection, the uterus began to firmly contract, gradually forcing out my hand with large masses of clotted blood, coagulated by the iron. I watched the patient for two hours, a half hour of the time firmly holding the uterus with my hand, and little or no hemorrhage occurred after the injection. She was relieved from the condition of collapse by brandy and hypodermic injections of atropia, and made a rapid recovery without any bad symptoms. In this case, expecting a hemorrhage, I gave her from the time the os was sufficiently dilated to admit of the passage of the child's head until the head was delivered two hypodermic injections of ergot, ʒj each, without avail. I had no time to try other expedients, but resorted at once to the iron.

CASE II. Mrs. K——, æt. 26, rather nervous temperament, sent for me on the morning of March 18, to attend her in her second confinement. Having attended her in the first labor, which was followed by hemorrhage that could only be controlled by a strong current of galvanism, I concluded I would be prepared to meet any emergency with the perchloride of iron. While the first stage of labor was progressing, I sent to the druggist for ℥ vi of the tr. perchlor. and added one-half of this to oj. of water. A Davidson syringe was made ready for use, and I then waited the dreaded hour. The labor became tardy and protracted, and she was delivered, after a long traction with the forceps, of a large male child. Ergot was given hypodermically twice, and kneading constantly kept up by the nurse during delivery. Hemorrhage at once set in and I attempted to control it by the introduction of ice into the uterus and grasping it with one hand on the abdomen, the other in the vagina. It was of no avail. The hemorrhage increased. I next tried the plan of irritating the interior of the uterus with the finger nails, but in doing this I completely detached the placenta, which was removed, followed by a hemorrhage which soon put my patient into a state of collapse. I at once began the injection of the solut. of ferri perchlor. into the cavity of the uterus, with the effect of immediately producing firm contraction and cessation of hemorrhage. She finally recovered after a mild attack of metritis, and to-day both she and child are doing well.

These constitute my experience in the use of this agent, but can any one doubt that the lives of these two women were saved by it? That there may be danger in its use I do not deny. While the uterus is in this relaxed condition, the mouths of the veins open, it may pass into them, causing clotting of blood, thereby producing thrombosis; but, notwithstanding this, I presume, from my own experience, as well as from the experience of the many able writers on this subject, that this mode of treatment in puerperal hemorrhage is not only justifiable, but under many circumstances strongly indicated.—*Med. Chron.*

